# Clinical challenges in the dosing and titration of cariprazine

**DOI:** 10.3389/fpsyt.2024.1427482

**Published:** 2024-08-30

**Authors:** Čedo D. Miljević, Petar G. Vuković, Ana Munjiza-Jovanović

**Affiliations:** ^1^ Clinic for Adults, Institute of Mental Health, Belgrade, Serbia; ^2^ Department of Psychiatry, Faculty of Medicine, University of Belgrade, Belgrade, Serbia; ^3^ Clinic for Children and Adolescents, Institute of Mental Health, Belgrade, Serbia

**Keywords:** cariprazine, antipsychotics, psychopharmacology, cross-titration, partial D3 agonist

## Abstract

The introduction of a new psychopharmaceutical medication instead of the previous one always poses a certain challenge. In the case of antipsychotics (AP), these problems are considerably more complicated and are mainly caused by the question of dose equivalents, but also by the pharmacokinetic properties of the drug. In the case of partial dopamine D2 agonists, an additional issue is the possibility of deterioration when switching from the previous D2 antagonists to these drugs. Cross-titration is therefore generally recommended. Finally, due to the capsule form, it is not possible to increase the dose of cariprazine by less than 1.5 mg during titration. In this paper, we have presented our proposal to replace the most commonly used second-generation APs with the third-generation AP cariprazine. We have taken into account the dose equivalents, the pharmacological forms of the drugs and their pharmacokinetic and pharmacodynamic properties.

## 1 Introduction

Cariprazine is a new antipsychotic (AP) on the market in the Republic of Serbia. It was originally patented by the pharmaceutical company Gedeon Richter in 2006. It was introduced into clinical use in September 2015 when it was approved by the US Food and Drug Administration (FDA) for the treatment of schizophrenia and acute mania and mixed episodes associated with bipolar disorder in adults. Since May 2019, it has also been approved for use in bipolar depression (1). Finally, at the end of 2022, it was approved for use in major depressive disorder (MDD) in patients over the age of 18 as an adjunctive therapy (2). Although cariprazine is a new member of the partial dopamine D2 agonist group (along with the well-known aripiprazole and the new AP brexpiprazole), its mechanism of action is far more complex. First of all, cariprazine is the only AP with a much higher affinity for D3 than for D2 receptors (3, 4). D3 autoreceptors, which have the highest affinity for dopamine, are thought to play an important role in responding to the slow, irregular tonic activity of dopaminergic neurons by balancing the amplitude of a dopaminergic burst during rapid phasic activity - disruption of this balance is thought to have important implications for the pathophysiology of schizophrenia in all major symptom domains (5, 6). Furthermore, cariprazine is not only a partial agonist at the level of D3 and D2 receptors, but also a partial agonist of serotonin 5-HT1A receptors and an antagonist of serotonin 5-HT2B receptors. Remarkably, its affinity for D3 receptors is approximately 1000 times greater than that of dopamine, while its affinity for D2 receptors is approximately 100 times greater than that of dopamine (7, 8). Considering the receptor binding profile of cariprazine and the preclinical studies demonstrating its efficacy in improving not only positive (9), but also negative symptoms (10) and cognitive deterioration (9, 11) in various behavioral animal models of schizophrenia, cariprazine is a very interesting molecule with a potentially broad application in various psychotic disorders, especially schizophrenia and schizophrenia-like disorders.

## 2 Efficacy and safety of cariprazine in clinical studies

Previous clinical studies have largely confirmed the above, and it is now widely believed that cariprazine is not only effective in the treatment of acute schizophrenia, but is also the first AP to be superior to other APs (12) in treating the predominant negative symptoms of schizophrenia [which are significant predictors of poorer functional outcomes in patients with schizophrenia (13)]. Finally, clinical studies have shown its clear efficacy in the treatment of manic and mixed episodes of bipolar disorder (14–16) and, more recently, in the treatment of depressive episodes of bipolar disorder (17–19) and as adjunctive therapy in MDD (20).

The most common side effects of cariprazine in clinical trials in patients with schizophrenia included akathisia and extrapyramidal symptoms, while it showed a neutral metabolic profile and weight gain was clinically insignificant compared to placebo (1, 21). On the other hand, cariprazine shows positive effects on prolactin (it has the potential to lower prolactin levels that were previously elevated by the action of another AP) (22).

## 3 Pharmacokinetics of cariprazine

The half-life of cariprazine is about 2-4 days and that of the active (equipotent) metabolites desmethyl-cariprazine (DCAR) and didesmethyl-cariprazine (DDCAR) is 1-2 days and 1-3 weeks, respectively (1). The distribution equilibrium of cariprazine and DCAR is established in 1-2 weeks, while the equilibrium of the total drug mixture is established in about 3 weeks (due to the very long half-life of the DDCAR metabolite) (23). The major metabolic pathway is represented by the hepatic isoenzyme CYP3A4 (24).

## 4 Starting treatment with cariprazine - recommendations and considerations

As with all medications, switching therapy and replacing one medication with another requires special attention and strategy. Although there are certain clear recommendations for cariprazine (25, 26), the situation in daily clinical work is not free of certain problems, considerations and dilemmas.

First of all, it is now generally considered that there are two methods of introducing cariprazine: the fast and the slow method. In the rapid method, cariprazine is introduced at a dose of 1.5 mg and the dose is then increased by 1.5 mg each day to the maximum allowable dose of 6 mg/day. Variations of this approach are that the dose is increased every third or fifth day (27–29). Rapid initiation of treatment with cariprazine is recommended in patients with a florid clinical picture. In these patients, improvement has been observed in the first week at doses of 3, 4.5 or 6 mg/day (29). The slow method of introducing cariprazine has an advantage in daily work and involves increasing the dose of cariprazine by 1.5 mg per week up to a dose of 4.5 mg, which is reached after 2 weeks (12). This method of introducing cariprazine is recommended in stable patients, i.e. patients who do not have florid symptoms.

In addition, current recommendations for the introduction of cariprazine speak of the use of so-called cross-titration, i.e. the dose of the previous AP is gradually reduced and the dose of cariprazine is gradually increased until a dose of cariprazine is reached that corresponds to the dose of the previous AP.

And although this seems very simple and clear at first glance, unfortunately it is not. First of all, there is the question of an appropriate dose, i.e. dose equivalents. Another problem arises from the pharmacological form of cariprazine, i.e. cariprazine is available in capsules, which cannot be divided, but can only be taken as a whole (in a precisely defined dose). This raises the question of how much cariprazine the patient should receive, e.g. on day 3, 7 or 14 of cross-titration. Therefore, due to the capsule form, the smallest increase in cariprazine dose is only possible at 1.5 mg (this is the minimum effective dose of cariprazine).

## 5 Cariprazine dose equivalents

The question of an appropriate dose of cariprazine compared to the previously used APs is a question of dose equivalents. When considering the dose equivalents of second/third-generation APs, the first thing to bear in mind is that they should be taken with some caution. The mechanism of action of these APs differs considerably from that of first-generation APs. While first-generation APs exert their effects exclusively via antagonism of the dopamine D2 receptors (leading to the famous Seeman exponential curve of the relationship between the inhibitory concentration of first-generation APs (IC50) at the dopamine D2 receptors and their recommended daily dose) (30), second- and third-generation APs have a far more complex mechanism of action. This mechanism also includes effects on other receptors (especially serotonin), possibly via the different dissociation rates of these APs from the target receptors (31), as well as the already mentioned completely different effect on dopamine D2 receptors (partial agonism). Although the dopamine D2 receptors are similar in the different brain regions and the antipsychotics show similar penetration into the different brain regions (except the pituitary gland), it should also be noted that all measurements of pharmacodynamic parameters relevant to the mechanism of action of APs were performed in the dorsal striatum, which (apart from the side effects) is of completely secondary importance for the therapeutic effect of the APs (compared to e.g., cortex, nucleus accumbens or ventral tegmental area) (32).

From all this, it is clear that the calculation of dose equivalents, i.e., which dose of the newly introduced AP corresponds to the dose of the previously taken drug, is by no means simple, which is why there are numerous methods, and each of them has its shortcomings. Some of the most commonly used methods for determining dose equivalents are the Minimum Effective Dose method (32), the Mean Dose method (33), and the Defined Daily Dose method (DDD) (34). In a recently published meta-analysis of the dose-response relationship in AP therapy (35), another method was used to calculate dose equivalents based on the AP dose that results in 95% of total symptom reduction (the so-called ED 95 value). Finally, pharmacotherapy guides (secondary sources) are often used to suggest approximations for dose equivalents based on synthesizing data from multiple primary sources – for example, The Maudsley Prescribing Guidelines in Psychiatry (36). In **Table 1** we have provided dose equivalents for 1.5 mg cariprazine based on some of the most frequently cited sources (34–36).

**Table 1:** Dose equivalents for 1.5 mg cariprazine – Modified from references 34-36; *Data for clozapine dose equivalents by the dose–response method were based on only one non-randomised study with 48 subjects and should therefore be considered with caution; **Maudsley’s guideline does not recommend clozapine dose equivalents.

Antipsychotic	DDD method (34)	Dose – response method (35)	Maudsley recommendations (36)
Cariprazine	1.5mg	1.5mg	1.5mg
Olanzapine	5mg	3mg	5mg
Risperidone	2.5mg	1.2mg	1.5mg
Aripiprazole	7.5mg	2.25 mg	7.5mg
Clozapine	150mg	112.5mg*	/**
Quetiapine	200mg	94.8mg	150mg

DDD, the Defined Daily Dose method.

**Figure 1:**
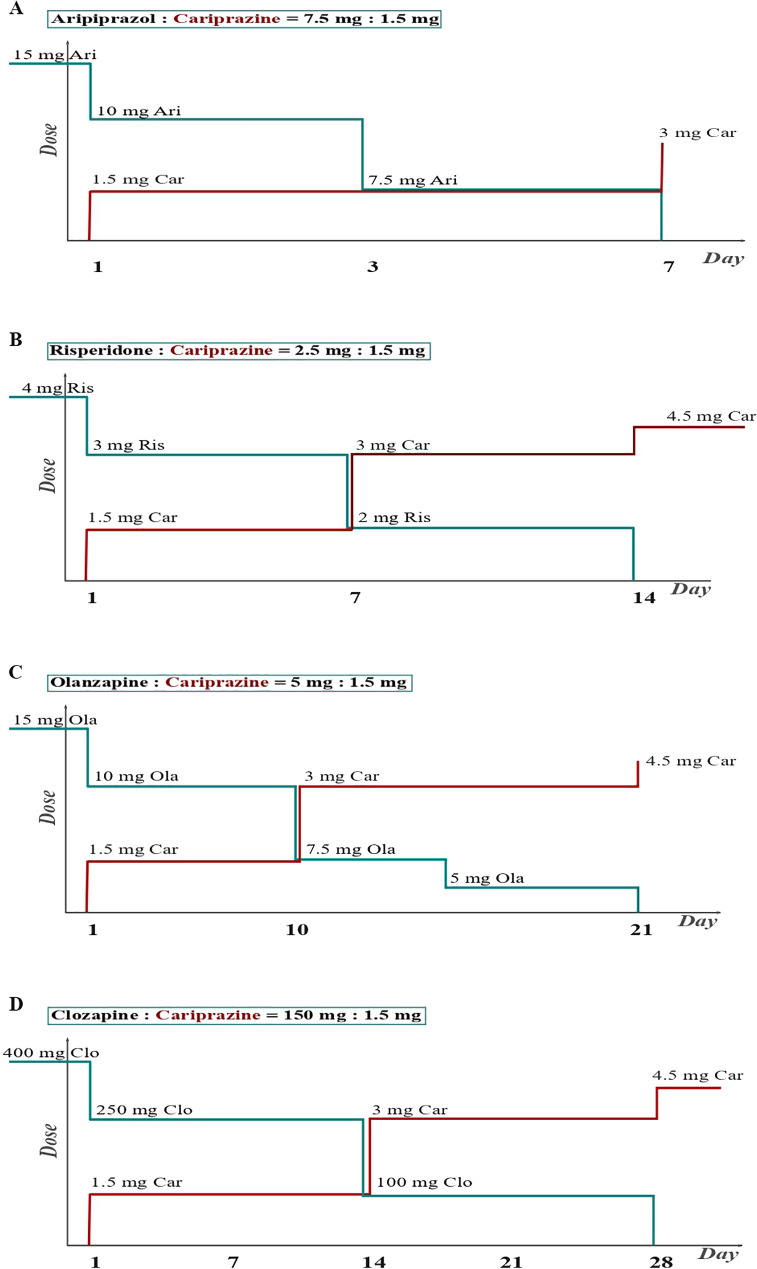
Switching the patient from second and third-generation antipsychotics to cariprazine. **(A)** Switching the patient from aripiprazole to cariprazine. When switching from an antipsychotic that has a similar receptor profile to cariprazine, i.e. a partial D2 agonist, a 1-week cross-titration is recommended. The previous antipsychotic should be discontinued within 1 week, while the cariprazine dose is introduced and increased within the same week (26). **(B)** Switching the patient from risperidone to cariprazine. When switching from a second-generation antipsychotic that exerts D2 antagonism, about two weeks are required. This is necessary to avoid a dopaminergic rebound, which leads to increased psychotic symptoms, agitation, and dyskinesia (26). **(C)** Switching the patient from olanzapine to cariprazine. The longest time is required when switching from antipsychotics with a completely different receptor profile, such as antipsychotics with stronger antihistaminic/anticholinergic effects. Allowing sufficient time will avoid histaminergic and cholinergic rebound, reducing the risk of insomnia, nausea, and vomiting (26). **(D)** Switching the patient from clozapine to cariprazine. Based on US data from the field, a four-week cross-titration is recommended for clozapine. The four trajectories of the switch to cariprazine shown in the figure are theoretical models and do not represent specific patients.

What does this mean in practice? We will discuss four different scenarios - namely switching from aripiprazole, risperidone, olanzapine and clozapine to cariprazine. In the proposals presented, we have used the DDD method to determine dose equivalents (34). It should be noted that the four trajectories of switching to cariprazine discussed in the following text and illustrated in **Figure 1** are theoretical models and not data from actual patients. It is also important to point out that, to our knowledge, no study to date has specifically investigated the clinical efficacy, tolerability and safety of cariprazine after switching from current treatment with other antipsychotics - a finding confirmed by a recent review by Baumann and colleagues (37). However, in a pivotal study by Nemeth and colleagues comparing the efficacy of cariprazine with risperidone in patients treated for predominantly negative symptoms of schizophrenia, a slow cross-titration between 2 and 4 weeks was used for switching from the previous AP to cariprazine/risperidone (12). In addition, the recommendations of the European Medicines Agency (EMA) (38) and the International Panel on Cariprazine (39) indicate that a cross-titration method should be used when switching from a previous AP to cariprazine. In the absence of stronger evidence and to comply with currently available recommendations, a cross-titration method was used in the cases proposed below. The use of standardized, validated psychometric scales is recommended when switching between APs to assess treatment efficacy (e.g. Positive and Negative Syndrome Scale (40)) and tolerability (e.g. UKU scale (41)).

## 6 Switching models

### 6.1 Switching from second or third-generation antipsychotics to cariprazine

The first scenario, in which a patient is switched from aripiprazole to cariprazine (**Figure 1A**), is the simplest and requires the least amount of time. Since aripiprazole and cariprazine are drugs from the same subgroup of APs (partial agonists at dopamine receptors/third generation of APs) and as they have a similar receptor affinity for other (non-dopamine) receptors, there is no need to fear a cholinergic, histaminergic, adrenergic and/or dopaminergic rebound, which can result from a long-term blockade of these receptors (and the resulting upregulation). In this case, it is recommended to make switching within one week (26, 39). In our example, a cross-titration is initiated in a patient who previously took 15 mg of aripiprazole by reducing aripiprazole to 10 mg and simultaneously introducing cariprazine at a dose of 1.5 mg. On day 3, we reduced the aripiprazole dose to 7.5 mg, and finally, on day 7, we eliminated aripiprazole and increased cariprazine to a dose of 3 mg. It is important to note that we always try to keep the dose of both drugs at such levels that the occupancy of the dopamine receptors changes as little as possible. Since both cariprazine and aripiprazole are partial D2 agonists, it is questionable whether an abrupt switch should be made. However, apart from the recommendations above, which do not exclude aripiprazole, an abrupt switch could cause problems with tolerability - a rapid introduction of cariprazine at higher doses carries a higher risk of a patient developing akathisia and discontinuing treatment (38, 39). Titrating the dose over a longer period reduces the likelihood of patients developing akathisia and discontinuing treatment (38, 39).

Another scenario in which a patient is switched from risperidone to cariprazine (**Figure 1B**) is slightly more complicated. As risperidone exerts a strong dopamine receptor blockade, there is a potential risk of dopaminergic rebound (and worsening of psychotic symptoms) when switching to partial D2 agonists (42, 43). To avoid this scenario, cross-titration over two weeks is recommended in the case of risperidone (26, 39). In our example, if a patient is taking 4 mg risperidone, we first reduce the risperidone dose to 3 mg and simultaneously introduce cariprazine at the lowest dose of 1.5 mg (equivalent to 2.5 mg risperidone – according to the DDD method (34)). After one week, we reduce the risperidone dose to 2 mg and increase the cariprazine dose to 3 mg. Finally, after another week, we discontinue risperidone completely and maintain the cariprazine dose at 3 mg (alternatively, we further increase the dose to 4.5 mg if other sources are used to determine dose equivalents (35, 36)). This allows for gradual regulation at the dopamine receptor level and avoids dopaminergic excess. At this point, it should be noted that there are also sources that recommend switching from risperidone to cariprazine (partial D2 agonists in general) within just one week (44). In this scenario in particular, increasing the dose of cariprazine to 3 mg (after one week) would allow discontinuation of risperidone. Since partial D2 agonists have a very high affinity for D2 receptors and presumably occupy most receptors even at lower doses, it would theoretically make more sense to accelerate the upward titration to reach the appropriate dose.

In the third scenario, a patient is switched from olanzapine to cariprazine (**Figure 1C**). Certain second-generation APs (such as olanzapine, quetiapine, and asenapine) have a relatively high receptor affinity for histamine, muscarinic, and alpha-1-adrenoceptors. Since cariprazine has a low affinity for these receptors, additional caution and even more gradual cross-titration is required to avoid side effects resulting from histaminergic, adrenergic and/or cholinergic rebound in the newly created absence of blockade of (previously upregulated) receptors. In the above situation, cross-titration over 3 weeks is suggested (26, 39), as in the following example. In a patient taking 15 mg of olanzapine daily, we reduce the olanzapine dose to 10 mg and simultaneously administer 1.5 mg of cariprazine. After 7-10 days, we can safely reduce the dose of olanzapine further to 7.5 mg per day and increase cariprazine to the dose of 3 mg per day. Two weeks after starting cross-titration, we suggest reducing olanzapine to 5 mg per day while maintaining the cariprazine dose at 3 mg per day. Finally, on day 21, we can discontinue olanzapine and increase cariprazine to 4.5 mg.

The final scenario here is switching a patient from clozapine to cariprazine. General recommendations when discontinuing clozapine are that this process is carried out over several weeks (usually at least 4) (44) to avoid the effects of abrupt discontinuation mentioned earlier, which can be particularly pronounced with clozapine – the greatest risk of so-called “rebound” psychosis (45, 46). So, in our case (**Figure 1D**), we start with a patient taking 400 mg of clozapine daily with a dose reduction to 250 mg and simultaneously introducing 1.5 mg of cariprazine. After two weeks, we further increase the cariprazine to 3 mg, taking dose equivalence into account while simultaneously reducing the clozapine dose by a further 150 mg to 100 mg/day. Finally, on day 28, we excluded clozapine and increased the cariprazine dose to 4.5 mg, primarily out of a desire to avoid a possible “rebound” phenomenon. However, it is clear that the cariprazine dose of 3 mg is more appropriate according to the dose equivalents. In this way, through a very gradual titration, we avoid side effects caused by the upregulation of receptors while minimizing the risk of worsening psychotic symptoms.

### 6.2 Switching from cariprazine to second or third-generation antipsychotics

A recommendation should also be made to discontinue cariprazine, i.e., to switch from cariprazine to another AP. It is important to point out that due to the lack of more extensive clinical experience with cariprazine, the existing recommendations should still be considered with caution. As mentioned above, cariprazine (considering its active metabolites) has a long plasma half-life (about 3 weeks). With this in mind, some authors state that it is safe to discontinue cariprazine abruptly while gradually introducing another AP (25). If the patient is switched to a drug that acts on a broader range of receptors (olanzapine, quetiapine, asenapine), a gradual introduction (about 3 weeks) is recommended to acclimatize to the side effects of blocking these receptors (e.g. sedation, anticholinergic effects, orthostatic hypotension, etc.) (25).

## 7 Discussion and conclusion note

Cariprazine showed consistent efficacy compared to placebo across outcomes and was generally well tolerated, suggesting efficacy for the treatment of bipolar disorder – depression and manic state (18), both negative and positive symptoms in the first episode or relapse of schizophrenia (28, 47), and major depressive disorder (48). Even more, the results of some studies suggest that cariprazine was superior to another second-generation antipsychotic in the treatment of predominant negative symptoms. For example, the study by Németh and colleagues showed that cariprazine treatment was more effective than risperidone in the improvement of predominant negative symptoms in patients with schizophrenia (12). Another study also showed that cariprazine treatment versus risperidone made significant improvements in the treatment of negative symptoms of schizophrenia (49). Advantages of cariprazine were also observed for some populations of patients compared to antipsychotics of third generation. For example, Earley and coauthors highlighted results that in patients with acute schizophrenia and moderate/severe negative symptoms, cariprazine was associated with significantly greater improvement in negative symptoms compared with placebo and aripiprazole (50). Although we don’t have enough relevant research regarding a comparison of cariprazine vs. olanzapine оr clozapine treatment - cariprazine is usually better tolerated than olanzapine or clozapine primarily in terms of metabolic side effects (39). Adjunctive cariprazine treatment is also suggested to have a role in the treatment of „treatment-resistant’’ patients (51), and recent data suggest the efficiency of cariprazine combined with olanzapine or clozapine in improving patient’s positive symptoms, negative symptoms, and overall functioning (52, 53). In line with the augmentation effects of cariprazine, a notable number of research and meta-analyses indicate that the introduction of cariprazine as augmentation therapy in depression significantly reduces depressive symptoms compared to placebo or other AP (54, 55). Also, it’s important to emphasize that the side effects of cariprazine are dose-related, and most studies showed that at a dose of 1.5 mg/day cariprazine, as augmentation agent, had the most robust efficacy and good safety.

With all analyzed above it’s clear that cariprazine as a new partial agonist of the D2/D3 receptors has demonstrated efficiency across different clinical domains in clinical trials and studies, and that it’s a promising drug for many endophenotypes. With adequate titration and/or switching mechanisms, it could improve or treat a broad spectrum of disorders.

## Data Availability

The original contributions presented in the study are included in the article/supplementary material. Further inquiries can be directed to the corresponding author.
